# Geospatial and temporal mapping of detectable HIV-1 viral loads amid dolutegravir rollout in KwaZulu-Natal, South Africa

**DOI:** 10.1371/journal.pgph.0003224

**Published:** 2024-05-28

**Authors:** Lilishia Gounder, Andrew Tomita, Richard Lessells, Sandrini Moodley, Kerri-Lee Francois, Aabida Khan, Melendhran Pillay, Sontaga C. Manyana, Subitha Govender, Kerusha Govender, Pravi Moodley, Raveen Parboosing, Nokukhanya Msomi, Frank Tanser, Kogieleum Naidoo, Benjamin Chimukangara

**Affiliations:** 1 Department of Virology, Inkosi Albert Luthuli Academic Complex, National Health Laboratory Service, Durban, South Africa; 2 Department of Virology, School of Laboratory Medicine and Medical Sciences, University of KwaZulu-Natal, Durban, South Africa; 3 Centre for Rural Health, School of Nursing and Public Health, University of KwaZulu-Natal, Durban, South Africa; 4 KwaZulu-Natal Research Innovation and Sequencing Platform (KRISP), College of Health Sciences, University of KwaZulu-Natal, Durban, South Africa; 5 Centre for the AIDS Programme of Research in South Africa (CAPRISA), Durban, South Africa; 6 South African Medical Research Council (SAMRC), CAPRISA HIV-TB Pathogenesis and Treatment Research Unit, Durban, South Africa; 7 School of Pathology, University of Witwatersrand & National Health Laboratory Service, Johannesburg, South Africa; 8 Africa Health Research Institute, KwaZulu-Natal, Durban, South Africa; 9 School of Nursing and Public Health, University of KwaZulu-Natal, Durban, South Africa; 10 Centre for Epidemic Response and Innovation, School for Data Science and Computational Thinking, Stellenbosch University, Stellenbosch, South Africa; 11 DSI-NRF Centre of Excellence in Epidemiological Modelling and Analysis (SACEMA), Stellenbosch University, Stellenbosch, South Africa; 12 Critical Care Medicine Department, NIH Clinical Center, Bethesda, Maryland, United States of America; GU: Georgetown University, UNITED STATES

## Abstract

South Africa rolled out dolutegravir (DTG) as first-line antiretroviral therapy (ART) in December 2019 to overcome high rates of pretreatment non-nucleoside reverse transcriptase inhibitor drug resistance. In the context of transition to DTG-based ART, this study spatiotemporally analysed detectable HIV viral loads (VLs) prior to- and following DTG rollout in public-sector healthcare facilities in KwaZulu-Natal (KZN) province, the epicentre of the HIV epidemic in South Africa. We retrospectively curated a HIV VL database using de-identified routine VL data obtained from the National Health Laboratory Service for the period January 2018 to June 2022. We analysed trends in HIV viraemia and mapped median log_10_ HIV VLs per facility on inverse distance weighted interpolation maps. We used Getis-Ord Gi* hotspot analysis to identify geospatial HIV hotspots. We obtained 7,639,978 HIV VL records from 736 healthcare facilities across KZN, of which 1,031,171 (13.5%) had detectable VLs (i.e., VLs ≥400 copies/millilitre (mL)). Of those with detectable VLs, we observed an overall decrease in HIV VLs between 2018 and 2022 (median 4.093 log_10_ copies/mL; 95% confidence interval (CI) 4.087–4.100 to median 3.563 log_10_ copies/mL; CI 3.553–3.572), p<0.01 (median test). The downward trend in proportion of HIV VLs ≥1000 copies/mL over time was accompanied by an inverse upward trend in the proportion of HIV VLs between 400 and 999 copies/mL. Moreover, specific coastal and northern districts of KZN had persistently higher VLs, with emergent hotspots demonstrating spatial clustering of high median log_10_ HIV VLs. The overall decrease in HIV VLs over time shows good progress towards achieving UNAIDS 95-95-95 targets in KZN, South Africa. The DTG-transition has been associated with a reduction in VLs, however, there is a need for pre-emptive monitoring of low-level viraemia. Furthermore, our findings highlight that specific districts will need intensified HIV care despite DTG rollout.

## Introduction

The commitment to end the global AIDS epidemic by 2030, albeit shadowed by the COVID-19 pandemic, must be accompanied by accelerated action in upscaling effective HIV services [1]. Although the UNAIDS 95-95-95 strategy aims at ensuring that 95% of all people living with HIV (PLWH) are diagnosed, 95% of all PLWH diagnosed receive antiretroviral therapy (ART), and 95% of people on ART achieve sustained viral suppression, South Africa is still lagging behind in achieving these targets [1–4]. In KwaZulu-Natal (KZN), the province with the greatest burden of HIV in South Africa [5, 6], there are HIV-hyperendemic areas where sustained viral suppression among PLWH is substantially lower (53.9%) than the UNAIDS target [7]. Studies have shown that population prevalence of detectable viraemia could infer the effectiveness of HIV treatment programmes as well as community HIV risk potential [8–10].

Critical to the success of ART programmes is HIV viral load (VL) monitoring, and a sustained VL <50 copies/millilitre (mL) of plasma is considered treatment success [11]. HIV VL testing is recommended every 6–12 months, with annual VL monitoring in patients with viral suppression at follow-up [12]. Over the years, virological failure has been defined as two consecutive VLs ≥1000 copies/mL, 3 months apart, in patients on ART for at least 6 months. However, patients receiving first-line dolutegravir (DTG)-based ART are only considered for second-line ART when they have at least two VLs ≥1000 copies/mL after 24 months of DTG-based treatment, or have other signs of immunologic or clinical failure [13, 14]. While a plasma VL between 200 and 500 copies/mL significantly predicts future virological failure [15], VLs between 400 and 999 copies/mL are associated with an increased 10-year risk of death [16].

Routine HIV VL testing is only beneficial if accompanied by appropriate and timely clinical action, especially since prolonged treatment regimen failure significantly increases the risk of multiple HIV drug resistant mutations and compromises the efficacy of second-line ART in PLWH [17]. Challenges to VL monitoring of patients on ART include lack of infrastructure and poor service delivery, particularly in rural communities [18]. Moreover, facility-based VLs do not necessarily correlate well with population-based VLs and may not be predictive of HIV incidence [9]. Technologies, such as spatial information management systems with accurate HIV and infrastructure data, could play a significant role in improving the quality of care for PLWH and enhancing accessibility to healthcare services [19, 20].

The advancement of spatial statistics has allowed the application of disease mapping and spatial analyses in epidemiological research [21]. Geographic information systems (GIS) and spatial scan statistics have been applied within the context of the HIV epidemic in South Africa to help healthcare workers better understand levels of VL suppression [22] and HIV-related mortality patterns [21] in space and time. GIS has also been used as a means of visualizing the spread and transmission of diseases in South Africa. However, spatiotemporal tools have not yet been used to describe HIV VL monitoring in KZN with respect to changing treatment regimens [18]. We therefore aimed to describe spatiotemporal changes in HIV VLs in KZN, during the rollout and subsequent implementation of DTG in ART regimens as per the standard of care in South Africa.

## Methods

### Study design

This was a retrospective study of HIV VL data obtained from the South African National Health Laboratory Service (NHLS) Central Data Warehouse (CDW). The NHLS is the largest diagnostic pathology service provider in South Africa, delivering cost-efficient health laboratory services to all public-sector healthcare facilities that serve approximately 80% of the population [23]. The NHLS CDW stores data on laboratory testing with linked patient demographic data, which is vital for monitoring national and provincial Department of Health programmes [24].

**Fig 1:**
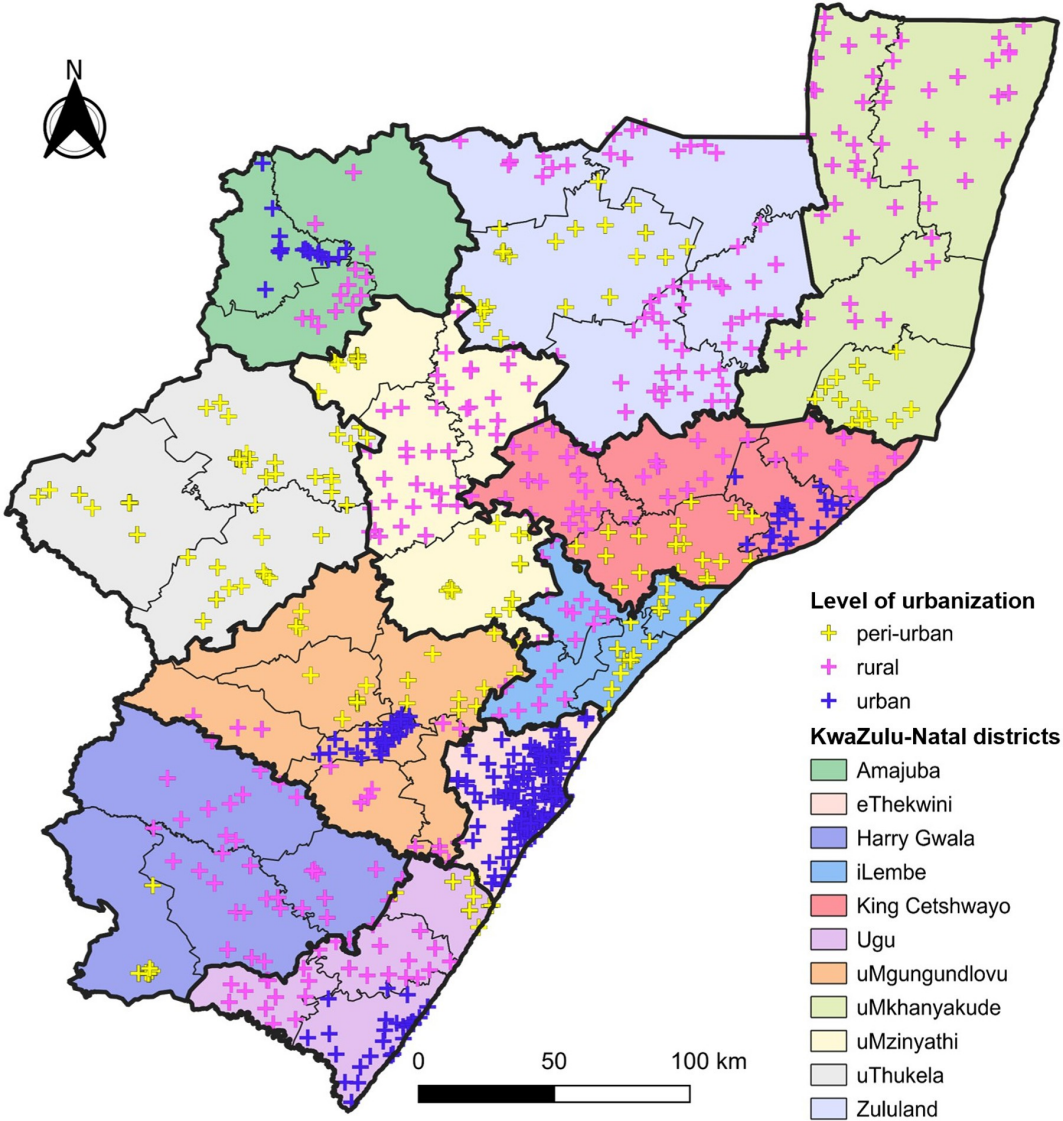
Healthcare facilities by district in KwaZulu-Natal province, South Africa. Cross symbols represent the location of each healthcare facility that had HIV VL data included in this study. The colour of the cross symbols denotes whether the facility location is within a peri-urban, rural or urban subdistrict. Districts are represented by individual colours. Thick and thin black outlines represent the borders of the districts and subdistricts, respectively. Republished from https://pinea.app.carto.com/map/4d4c56c1-f82d-4409-b190-ea9ced309005 under a CC BY license, with permission from Carto Builder user Lilishia Gounder, original copyright 2024.

We obtained de-identified data for all people with HIV who had HIV VLs between 1 January 2018 and 30 June 2022, at 736 public-sector healthcare facilities in KZN Province (Fig 1). This represents a period during the rollout and subsequent implementation of DTG in ART regimens in public-sector healthcare in South Africa. The anonymized data were accessed for research purposes from 21 September 2022 to 22 November 2023. All HIV VLs were tested at NHLS virology laboratories that actively subscribe to external quality assurance programmes (i.e., Quality Control for Molecular Diagnostics, Glasgow, Scotland, UK).

The automated molecular diagnostic analysers used for VL testing were the Abbott m2000 RealTime System (Abbott Molecular Diagnostics, Des Plaines, Illinois, United States), Abbott Alinity m System (Abbott Molecular Diagnostics, Des Plaines, Illinois, United States) and Roche Cobas 6800/8800 System (Roche Molecular Diagnostics, Pleasanton, California, United States). The limit of detection (LOD) varied depending on the analyser and the input volume used. For the Abbott m2000 RealTime System, the LODs were <40 and <150 copies/mL for 0.6 mL and 0.2 mL specimens, respectively. For the Abbott Alinity m System, the LODs were <20 and <50 copies/mL for 0.6 mL and 0.2 mL specimens, respectively. For the Roche Cobas 6800/8800 System, the LODs were <20 and <50 copies/mL for 0.5 mL and 0.2 mL specimens, respectively. In view of the variability in LOD, we defined detectable HIV VLs as any plasma VL ≥400 copies/mL and suppressed HIV VLs as any plasma VL <400 copies/mL.

### HIV viral load database

We created an in-house HIV VL database at the NHLS Department of Virology, Inkosi Albert Luthuli Central Hospital in Durban, South Africa, using de-identified HIV VL data obtained from the NHLS CDW. VL records originating from facilities outside of KZN were excluded from further analysis. Our analyses included records with suppressed VLs i.e., <400 copies/mL and detectable VLs, i.e., ≥400 copies/mL. We further categorized detectable VLs into two groups, i.e., (i) low-level viraemia; defined as any plasma VL 400–999 copies/mL and (ii) high VL; defined as any plasma VL ≥1,000 copies/mL. The database included the following meta-data for each VL record: age, sex, specimen collection date, KZN district, facility name and facility type. We assigned global positioning system (GPS) coordinates to each VL record based on facility location.

### Geospatial mapping

We generated maps with linked GPS coordinate locations from individual records in the HIV VL database using QGIS 3.30 software [25]. Each HIV VL record was represented by a linked GPS coordinate of the facility where the specimen was collected. The ratio of suppressed and detectable VLs was determined for each healthcare facility and used for geospatial mapping. We created inverse distance weighted (IDW) interpolation maps to map the ratio of suppressed VLs (i.e., VLs <400 copies/mL) and detectable VLs (i.e., VLs ≥400 copies/mL), over the study period.

To better understand spatiotemporal changes in levels of detectable HIV VLs (i.e., VLs ≥400 copies/mL) per facility, we used a deterministic spatial model (IDW interpolation) to interpolate the median log_10_ VLs in areas surrounding the healthcare facilities where the VLs were unknown. The method assumes that the median log_10_ VLs in populations within unsampled areas (i.e., areas surrounding the healthcare facilities) will correlate to the weighted average calculated between facilities. Areas closest to the healthcare facilities display median log_10_ VLs comparable to the actual median log_10_ VL measured at the neighboring facility location.

We used QGIS hotspot analysis 2.0 software [26] to determine spatial clustering (hot- or coldspot identification) of detectable HIV VLs. In summary, we applied local indicators of spatial association (LISA) statistics to perform Getis-Ord Gi* hotspot analysis [27], linking each statistical result to its respective facility location within the map layer. Using the Getis-Ord Gi* hotspot analysis values derived from both QGIS and Stata, we considered a facility to be in a hotspot if the p-value was <0.05 and z-score ≥+1.96. Similarly, we considered a facility to be in a coldspot if the p-value was <0.05 and z-score ≤-1.96.

We calculated spatial autocorrelation Moran’s I statistic using median log_10_ VLs per facility, and graphically represented index values for each year of the study period on Moran scatterplots using Stata 18.0 software. Moran’s I statistic measures spatial autocorrelation and index values for Moran’s I statistic range from -1 to +1. A positive index value indicates positive spatial autocorrelation. Positive spatial autocorrelation means that the median log_10_ VLs tend to be similar for facilities geographically nearby each other, i.e., high values located near high values and low values located near low values.

### Statistical analysis

HIV VLs were log_10_ transformed and reported as median VLs with interquartile ranges (IQRs) and or 95% confidence intervals (CIs). The median test was used to compare median VL across demographic groups and year of specimen collection. The Cochrane-Armitage test was used to assess the overall trend in proportion VL ≥1000 copies/mL over the study period. We used the Jonckheere–Terpstra test to assess the trend in log_10_ VL over the study period, for records with VLs ≥400 copies/mL. All statistical analyses were performed using Stata 18.0 software SE (StataCorp. 2023, College Station, Texas, United States).

### Ethical considerations

This study was approved by the Biomedical Research Ethics Committee of the University of KwaZulu‐Natal (reference number: BREC/00003120/2021). We obtained additional permission from the NHLS Academic Affairs and Research Management System to use routinely processed retrospective de-identified HIV VL records for research purposes. This study did not request informed patient consent because anonymized data was used.

## Results

**Fig 2:**
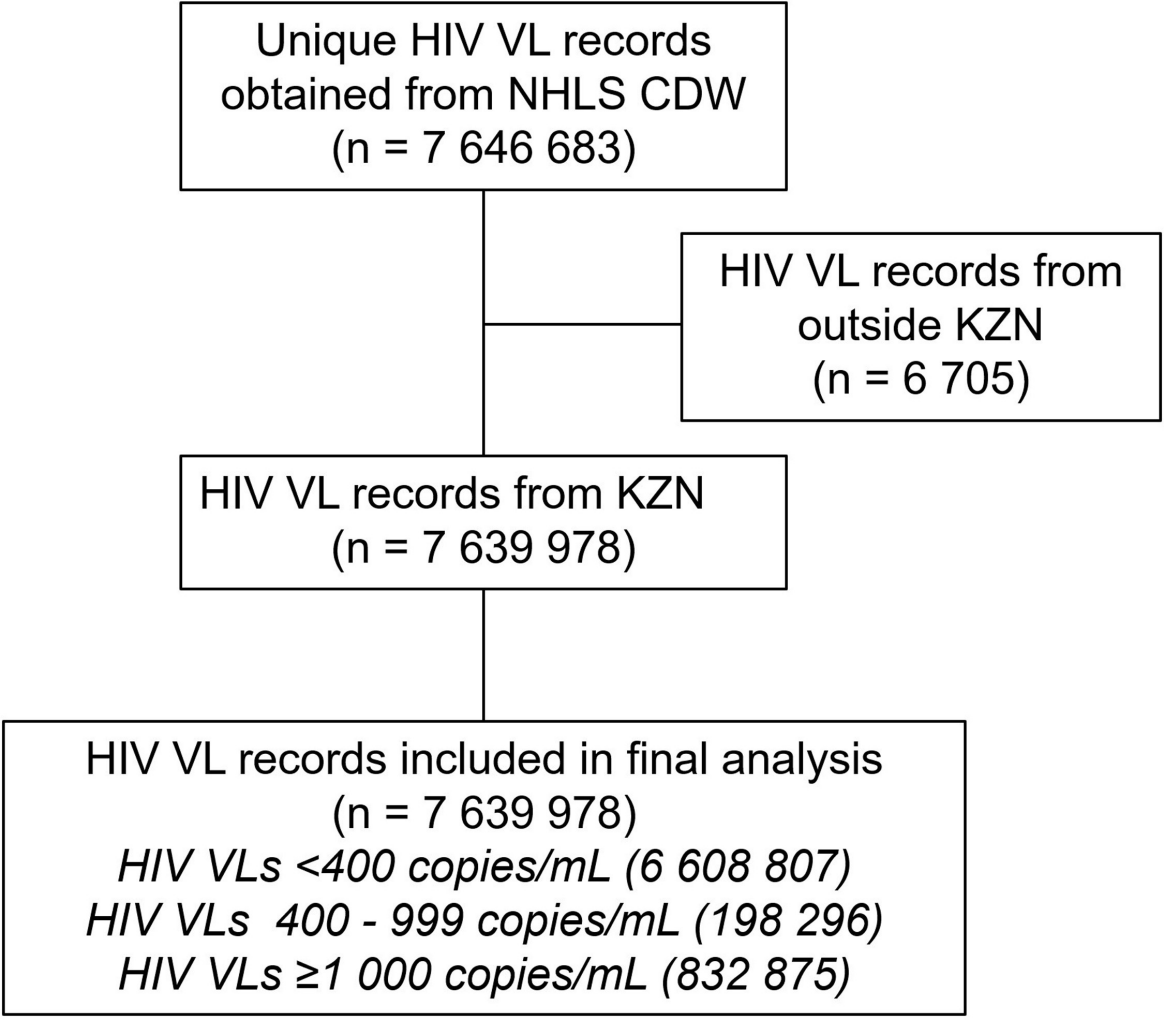
Flow diagram of HIV VL records obtained and included in final analysis. CDW, central data warehouse; HIV VL, HIV viral load; KZN, KwaZulu-Natal; mL, millilitre; NHLS, National Health Laboratory Service.

In total we curated 7,639,978 HIV VL records from 736 healthcare facilities in KZN. The majority of VLs (7,273,492; 95.2%) were from outpatient departments or primary healthcare facilities, with a smaller proportion (333,743; 4.4%) coming from inpatient wards and emergency departments, as well as patients institutionalized in correctional facilities and frail care centres (32,743; 0.4%). Approximately half (3,941,234/ 7,639,978; 51.6%) of all VL records were from facilities within urban subdistricts, with smaller proportions from rural (1,943,486; 25.4%) and peri-urban (1,755,258; 23.0%) subdistricts. Fig 2 shows a summary of HIV VL records obtained and analysed.

**Fig 3:**
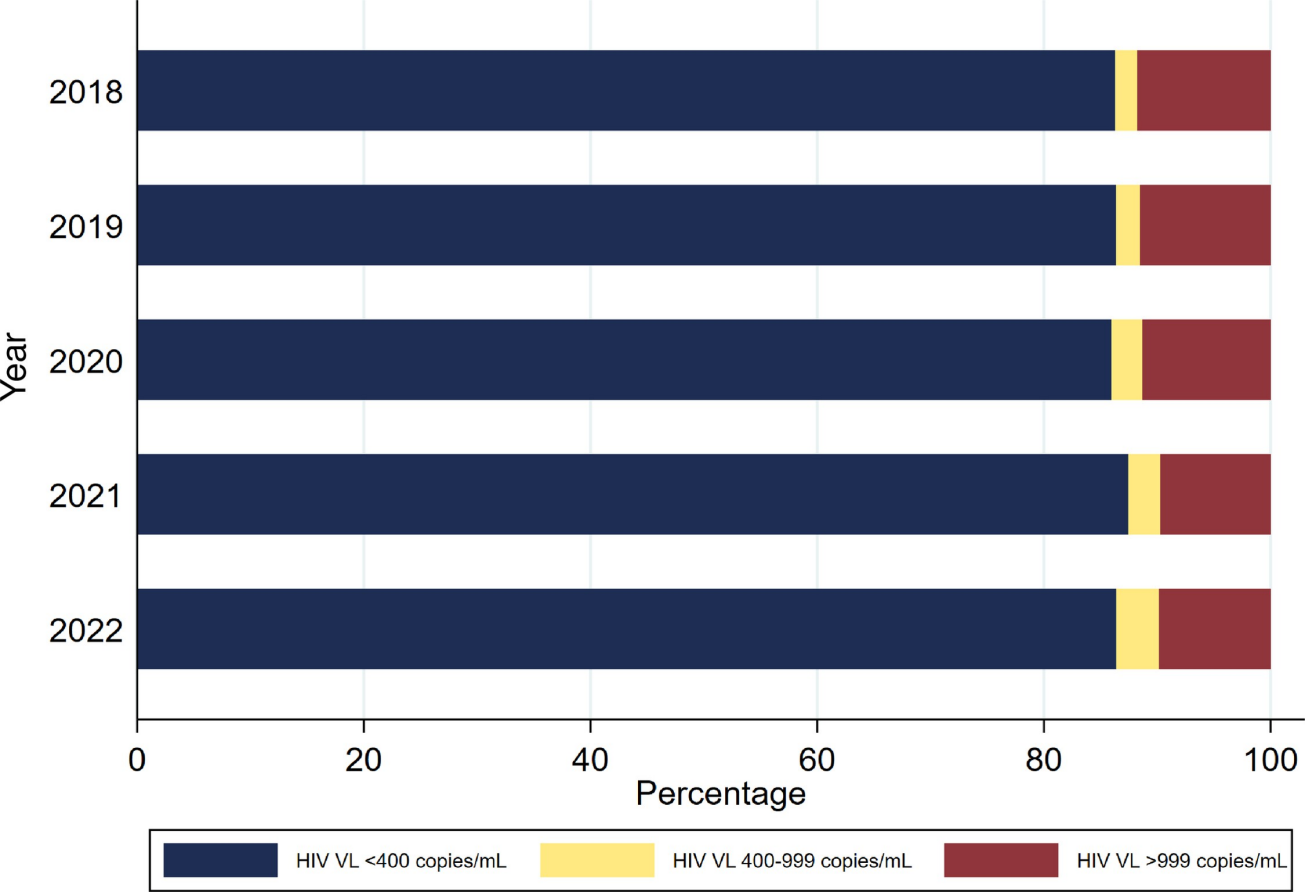
Proportion of all HIV viral loads by year in KwaZulu-Natal province, South Africa. mL, millilitre; VL, viral load.

The overall proportion of VLs from patients who had achieved VL suppression (<400 copies/mL) was 86% in the period 2018 to 2020, increasing to 87% in 2021 and returning to 86% in 2022. Females had higher VL suppression rates (4,579,540/ 5,209,487; 87.9%) than males (1,834,681/ 2,204,485; 83.2%). The average proportion of VLs ≥1000 copies/mL decreased significantly from 12% in the period 2018 to 2019, to 11% in 2020, with a further reduction to 10% in 2021 and 2022, p<0.05 Cochrane-Armitage test. The average proportion of VLs from patients who had low-level viraemia (400–999 copies/mL) was 2% in 2018 and 2019, increasing to 3% in 2020 and 2021, and up to 4% in 2022 (Fig 3).

The overall proportions of VLs per subdistrict were heterogeneous, with fluctuating trends noted during the period from 2018 to 2022 (S1 Table). We observed a general trend of increasing VL suppression rates between 2018 and 2022, with high ratios of suppressed to unsuppressed VLs. Despite the overall increase in VL suppression rates over time, there was a notable decrease in the proportion of suppressed VLs in northern KZN by 2022 (S1 Fig). Table 1 shows characteristics of all patients with HIV VLs, categorized by the level of HIV-1 viraemia (i.e., <400 copies/mL, 400–999 copies/mL and ≥1000 copies/mL).

**Table 1:** Characteristics of database records by level of HIV-1 viraemia.

Variable	All VL (n = 7 639 978)	VL <400 copies/mL (n = 6 608 807)	VL 400–999 copies/mL (n = 198 296)	VL ≥1000 copies/mL (n = 832 875)
**Sex**				
Female	5 209 487 (68.19%)	4 579 540 (69.29%)	128 476 (64.79%)	501 471 (60.21%)
Male	2 204 485 (28.85%)	1 834 681 (27.76%)	63 996 (32.27%)	305 808 (36.72%)
Unknown	226 006 (2.96%)	194 586 (2.94%)	5 824 (2.94%)	25 596 (3.07%)
**Age** in years, median (IQR)	37 (29–45)	37 (30–46)	36 (27–44)	33 (24–41)
<5	37 833 (0.50%)	21 262 (0.32%)	2 057 (1.04%)	14 514 (1.74%)
5–14	222 266 (2.91%)	146 383 (2.21%)	12 055 (6.08%)	63 828 (7.66%)
15–24	710 242 (9.30%)	539 469 (8.16%)	25 468 (12.84%)	145 305 (17.45%)
25–49	5 404 631 (70.74%)	4 750 906 (71.89%)	129 445 (65.28%)	524 280 (62.95%)
>50	1 210 106 (15.84%)	1 105 594 (16.73%)	27 686 (13.96%)	76 826 (9.22%)
Unknown	54 900 (0.72%)	45 193 (0.68%)	1 585 (0.80%)	8 122 (0.98%)
**Collection year**				
2018	1 518 316 (19.87%)	1 309 792 (19.82%)	29 838 (15.05%)	178 686 (21.45%)
2019	1 640 031 (21.47%)	1 416 364 (21.43%)	34 740 (17.52%)	188 927 (22.68%)
2020	1 749 167 (22.89%)	1 503 704 (22.75%)	47 492 (23.95%)	197 971 (23.77%)
2021	1 769 600 (23.16%)	1 547 152 (23.41%)	49 888 (25.16%)	172 560 (20.72%)
2022 ^a^	962 864 (12.60%)	831 795 (12.59%)	36 338 (18.33%)	94 731 (11.37%)
**Healthcare department**				
Outpatients	7 273 492 (95.20%)	6 336 424 (95.88%)	186 822 (94.21%)	750 246 (90.08%)
Inpatients	333 743 (4.37%)	242 672 (3.67%)	10 949 (5.52%)	80 122 (9.62%)
Other ^b^	32 743 (0.43%)	29 711 (0.45%)	525 (0.26%)	2 507 (0.30%)
**Level of urbanization** ^**c**^				
Rural subdistricts	1 943 486 (25.44%)	1 674 192 (25.33%)	49 834 (25.13%)	219 460 (26.35%)
Peri-urban subdistricts	1 755 258 (22.97%)	1 499 314 (22.69%)	53 172 (26.81%)	202 772 (24.35%)
Urban subdistricts	3 941 234 (51.59%)	3 435 301 (51.98%)	95 290 (48.05%)	410 643 (49.30%)

IQR, interquartile range; mL, millilitre; VL, viral load

^a^ Data are for half the year because study period ended on 30 June 2022

^b^ Records from correctional facilities or frail care facilities

^c^ Obtained from annual reports and integrated development plans for individual subdistricts

To further understand the spatiotemporal changes in detectable VLs (which could potentially impact ART), from this point onwards we only included VL records ≥400 copies/mL in downstream analyses. For the detectable VLs (i.e., ≥400 copies/mL), the median log_10_ VL was lower in outpatients (3.856 log_10_ copies/mL; IQR 3.121–4.678) than in inpatient and institutionalized patients (4.495 log_10_ copies/mL; IQR 3.508–5.353), p<0.001 median test. Males had significantly higher median log_10_ VLs (4.097 log_10_ copies/mL; IQR 3.220–4.948) than females (3.809 log_10_ copies/mL; IQR 3.107–4.610), p<0.001 median test. The majority of detectable HIV VLs (904,625/ 1,031,171; 87.7%) were from primary healthcare clinics, community healthcare centres and district hospitals. Notably, nearly half (505,933/ 1,031,171; 49.1%) of all detectable HIV VLs were from patients attending healthcare facilities in urban districts, which includes eThekwini; the metropolitan municipality of KZN.

**Fig 4:**
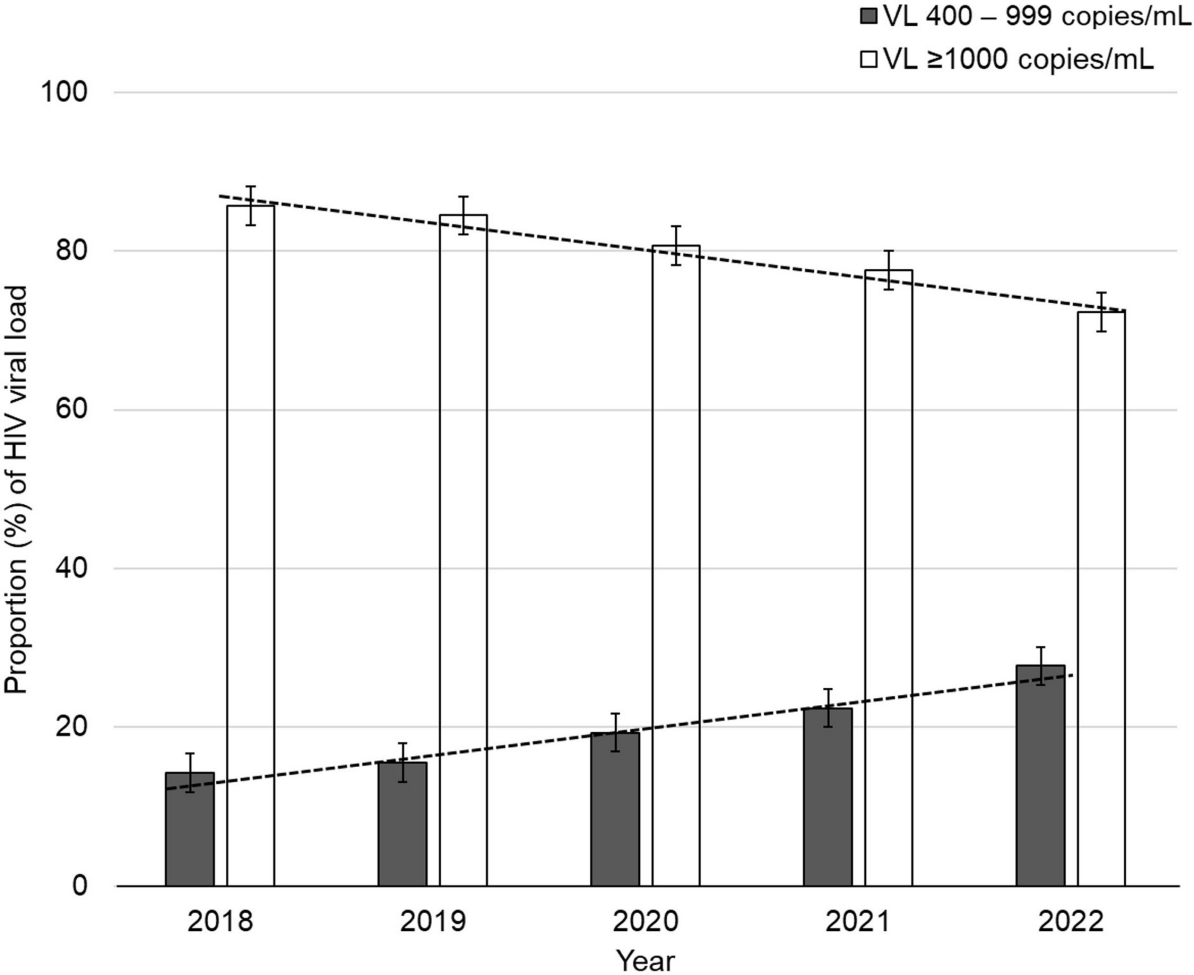
Trend analysis of detectable HIV viral loads by year in KwaZulu-Natal province, South Africa. mL, millilitre; VL, viral load.

There was a downward trend in the proportion of HIV VLs ≥1000 copies/mL between 2018 and 2022, with an inverse upward trend in the proportion of HIV VLs between 400 and 999 copies/mL (Fig 4). Overall, the median log_10_ VLs decreased significantly from 4.093 log_10_ copies/mL (CI 4.087–4.100) to 3.563 log_10_ copies/mL (CI 3.553–3.572) between 2018 and 2022, p<0.01 Jonckheere–Terpstra test (S2 and S3 Figs).

**Fig 5:**
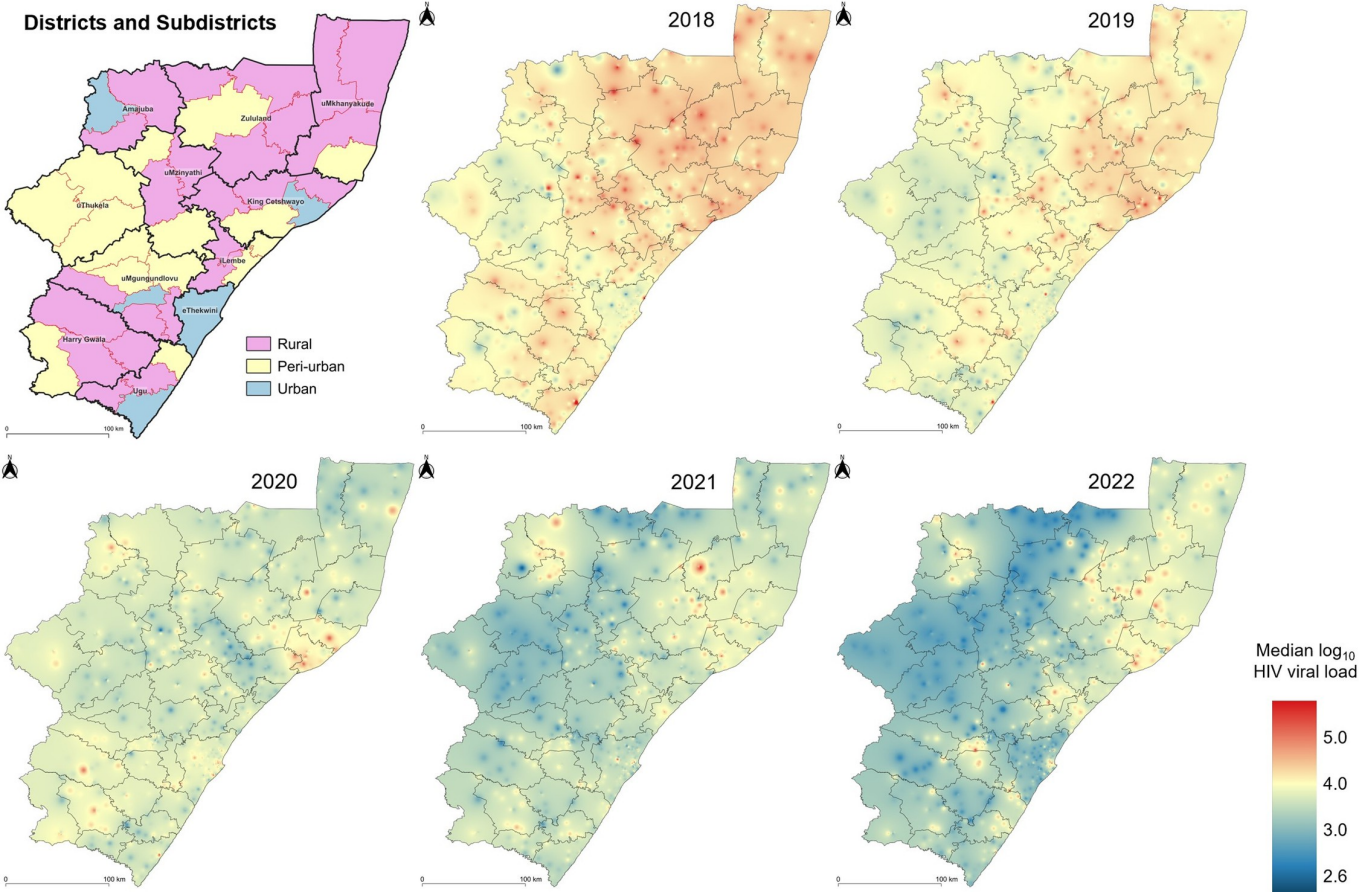
Decreasing HIV viral loads across subdistricts in KwaZulu-Natal province, South Africa. Inverse distance weighted interpolation maps from 2018 to 2022. Spectral colour change from red to blue reflects decrease in median log_10_ viral loads. Thick black outlines represent the borders of the 11 districts. Thin outlines represent the borders of the 44 subdistricts of KwaZulu-Natal. Republished from https://pinea.app.carto.com/map/4d4c56c1-f82d-4409-b190-ea9ced309005 under a CC BY license, with permission from Carto Builder user Lilishia Gounder, original copyright 2024.

We observed heterogeneous spatiotemporal changes in detectable HIV VLs per subdistrict, with most subdistricts reflecting a decrease in median log_10_ VL between 2018 and 2022 (Fig 5 and S4 Fig). Despite the overall reduction in HIV VLs over time, most districts along the coastal belt and in northern KZN maintained VLs ≥1000 copies/mL throughout the study period (S5 Fig).

**Fig 6:**
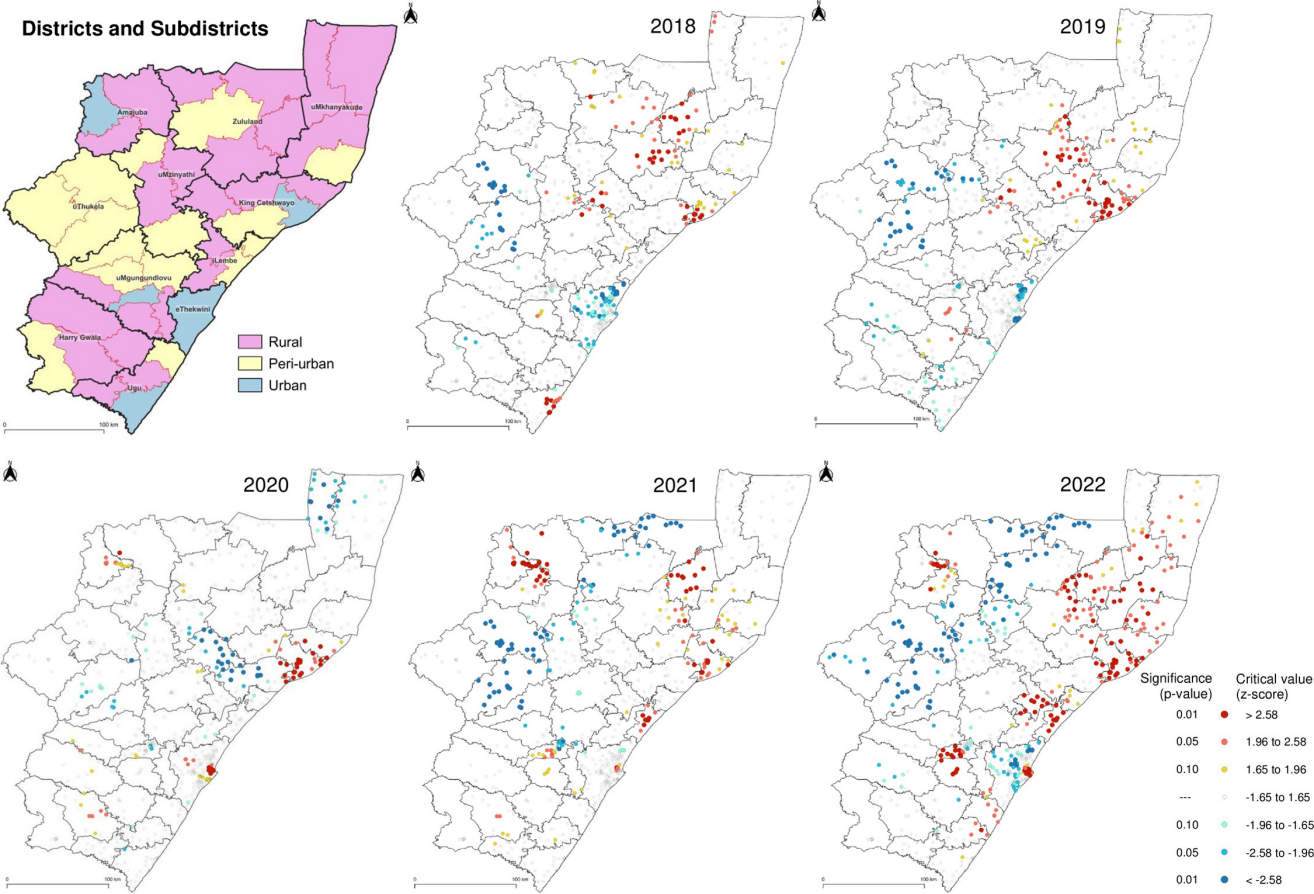
Hot- and coldspot analysis of HIV viral loads per facility in KwaZulu-Natal province, South Africa. Getis-Ord Gi* hot- and coldspot statistical analysis of median log_10_ viral load mapped per facility. Red, orange and yellow circles reflect the statistical significance of primary, secondary and tertiary hotspots at 99%, 95% and 90% confidence intervals, respectively. Light to dark blue circles reflect the statistical significance of tertiary, secondary and primary coldspots at 90%, 95% and 99% confidence intervals, respectively. Thick and thin outlines represent the borders of districts and subdistricts, respectively. Republished from https://pinea.app.carto.com/map/4d4c56c1-f82d-4409-b190-ea9ced309005 under a CC BY license, with permission from Carto Builder user Lilishia Gounder, original copyright 2024.

Getis-Ord Gi* hot- and coldspot analysis measured the spatial clustering of high and low median log_10_ VLs per facility, from 2018 to 2022. The relationship between geographical location and median log_10_ VLs was evaluated using the critical value standard deviation (z-score) and statistical significance (p-value) calculated per facility. Primary (z-score < -2.58 or > +2.58), secondary (z-score ±1.96 to ±2.58) and tertiary (z-score ±1.65 to ±1.96) intensity clusters are shown in Fig 6. Moran’s I statistic was calculated for median log_10_ VLs per facility, and the index value from 2018 to 2022 was >0.15, p<0.001, indicating positive spatial autocorrelation (S6 Fig).

Overall, 244 significant hotspot facilities demonstrated spatial clustering of high median log_10_ VLs, p<0.05 Getis-Ord Gi* statistic. The coastal King Cetshwayo district had persistent spatial clustering of high median log_10_ HIV VLs for facilities within urban and peri-urban subdistricts, from 2018 to 2022. During the transition period to DTG (2020 to 2022), 58 facilities were identified as hotspots for at least 2 consecutive years. These hotspot facilities were located within the northern districts of Amajuba and Zululand, the coastal districts of uMkhanyakude, King Cetshwayo, iLembe, eThekwini and its neighbouring district, uMgungundlovu. Of note, the coastal Ugu district, which did not have significant hotspots from 2019 to 2021, demonstrated emergent HIV VL hotspots in 2022 (S2 Table).

From 2018 to 2022, we compared database records from facilities within hot- and coldspots demonstrating spatial clustering of statistically significant high and low median log_10_ HIV VLs (S3 Table). The median age was 33 years (IQR 24–41 years) and 34 years (IQR 25–43 years) for the hot- and coldspot groups, respectively. There was a marginal difference in the proportion of males within the hot- and coldspot groups (35.2% versus 35.6%), and females within the hot- and coldspot groups (61.3% versus 61.8%). The median log_10_ VL was 4.140 log_10_ copies/mL and 3.558 log_10_ copies/mL for the hot- and coldspot groups, respectively. The proportion of inpatients was higher in hotspot facilities versus coldspots, throughout the study period. Hotspot facilities were mostly located within urban subdistricts and coldspot facilities were predominantly in peri-urban subdistricts as shown in S3 Table.

## Discussion

This study demonstrated that geospatial mapping of VLs offers a very useful tool for targeted health systems strengthening activities to achieve the UNAIDS 95-95-95 HIV treatment goals. More importantly this study highlights the need to direct efforts to known communities with higher levels of viraemia on ART, to strengthen VL re-suppression efforts and HIV prevention activities and ultimately, to implement activities to curb ART resistance emergence. This is especially important in resource-limited HIV-hyperendemic settings where strategic data-driven information, or epidemic intelligence can help appropriate deployment of scarce resources for maximal impact in epidemic control. We have shown that spatiotemporal mapping can be used to visually monitor VLs during a change in regimen. Furthermore, we have shown that spatiotemporal mapping is important in monitoring trends of viral suppression as well as identifying hotspots and geographical groups that require intensification of HIV services.

In our study, the majority of VLs were from outpatients attending public sector facilities that provide primary or level 1 healthcare. These findings support the WHO strategy that aims to strengthen the interface between primary healthcare and HIV services [28], highlighting the importance of HIV VL monitoring within primary healthcare settings. Infrequent monitoring of VLs, results in delayed detection of ART failure and intervention, with resultant clinical deterioration and increased risk of HIV transmission [11]. Our study showed heterogeneity in the distribution of detectable VLs by level of urbanization across the 11 districts of KZN. At a provincial level, we noted an overall downward trend in the proportion of VLs ≥1000 copies/mL between 2018 and 2022, which is in keeping with the steady downward trend in the proportion of VLs ≥1000 copies/mL observed at a national level [29].

We also observed an inverse upward trend in the proportion of low-level viraemia from 2% in 2018 to 4% in 2022, as more people who previously had high-level viraemia start to achieve viral suppression on more potent DTG regimens. An upward trend of low-level viraemia was also noted in Uganda, from 2% in 2016 to 8.6% in 2020 [30], amid first-line DTG rollout in 2018. The 2022 Ugandan HIV prevention and treatment guidelines now promote active monitoring of patients for low-level viraemia using non-suppression cut-offs of >200 copies/mL or >400 copies/mL for plasma and DBS, respectively [31]. In the South African setting, a previous study showed that low-level viraemia of 400–999 copies/mL was associated with a fivefold increased risk of virological failure [32], while another study suggested that patients with DTG resistance could present with delayed VL suppression or low-level viraemia [33]. Thus, to ensure the ongoing durability of DTG use in our setting, it would be pragmatic to actively monitor low-level viraemia in PLWH.

Using interpolation methods to geospatially map median VLs per facility location, we found that facilities in the rural northern districts of KZN as well as the peri-urban and urban coastal districts of KZN maintained relatively higher HIV VLs. Getis-Ord Gi* hot- and coldspot analysis showed spatial clustering of high median log_10_ HIV VLs i.e., hotspot facilities in the northern KZN districts of Amajuba, Zululand and uMkhanyakude for at least two consecutive years amid transition to DTG. Northern KZN has a high burden of HIV and or sexually transmitted infections [3]. Additionally, the northern districts that share borders with neighbouring countries, have exposure to cross-border transport or truck routes and historically, migrants and mobile populations are at higher risk of HIV acquisition [34]. Furthermore, these populations generally come to know their HIV status long after they are infected, inadvertently play a significant role in the transmission of HIV [35–37], experience challenges with linking to or remaining in HIV treatment programmes and as a result, may develop viral non-suppression [37]. This is further supported by the decrease in viral suppression that we observed in Northern KZN (S1 Fig), highlighting the need to strengthen VL monitoring in this region.

The districts along the coastal belt that had emergent hotspot facilities in 2021 and 2022 were iLembe and Ugu, respectively, while the metropolitan eThekwini district which has access to the main Durban trade port, had emergent hotspot facilities from 2020 to 2022. These districts are known to have a high triple burden of HIV, tuberculosis and sexually transmitted infections [3], together with the neighbouring inland area of uMgungundlovu which demonstrated re-emerging hotspot facilities during 4 of the 5 years (Fig 6). King Cetshwayo, which has access to the Richard’s Bay trade port, had spatial clustering of high median log_10_ HIV VLs from 2018 to 2022, specifically within peri-urban and urban subdistricts. Despite substantial progress in HIV testing and treatment coverage in King Cetshwayo district, previous studies have shown that a ‘core-group’ of individuals with unsuppressed VLs in this district are less likely to get tested for HIV and initiate ART [38–40]. Moreover, trade ports have been shown to be spaces of HIV vulnerability in southern Africa [41].

Emerging hotspots are of much concern given the positive impact DTG is expected to have on reducing population HIV VLs, due to its high genetic barrier to resistance [42, 43]. However, the emerging hotspots observed between 2020 and 2022 could be attributed to temporary disruptions caused by the COVID-19 pandemic in HIV treatment services, largely leading to ART interruptions and lack of treatment adherence [44]. In addition, HIV differentiated service delivery could play a critical role in supporting uninterrupted ART and reducing avoidable contact with healthcare facilities during pandemics such as that experienced with COVID-19 [45]. However, before deploying interventions, there is a need to evaluate the current systems in place and establish sustainable strategies for improving or developing these systems. Several studies have indicated an urgent need to develop systems that can collect locally relevant data in a standardized manner, to create geospatial models that can be accurately applied to the HIV epidemic [46–48].

Geospatial and temporal mapping are an essential part of strengthening health systems, and may be particularly useful in resource-limited settings [19]. Evidence supports the application of geospatial analysis to the field of public health and the HIV epidemic [18, 49–52]. A multinational study in sub-Saharan Africa demonstrated the benefits of mapping HIV clusters as a strategy for identifying populations at high risk of HIV infection, while another study used the technology to identify correlations between migration patterns and risk of HIV acquisition [51, 52]. Other studies showed that GIS could be applied to modelling the usage and accessibility of healthcare facilities [18] as well as understanding the overlap between infectious and non-infectious diseases to provide multi-disease care targeted to specific populations in rural South Africa [53]. Although our study is a retrospective analysis, our database and geospatial methods could be explored further to build a system to geospatially monitor HIV VLs in real time, to inform health system interventions.

Our findings using geospatial and temporal analysis should be interpreted with consideration of the following limitations. This study is based on facilities, not on households, and it is presumed that individuals would visit the healthcare facility closest to their place of residence, which is not always the case. It has been shown, however, that two-thirds of South Africans live less than 2 km away from their nearest public clinic [54]. HIV VL testing practices may differ by facility or district, which could influence the results of this study. Also, a study conducted in one rural district in KZN suggested that community VL derived from population-based surveillance did not necessarily correlate with routine facility-based VL measurements [9]. We created a database of de-identified laboratory test data, not individual patient data, hence there was no accurate unique patient identifier to link patient data serially. The data represents only patients who had a VL test and may therefore underestimate VLs in this population, as those lost to follow-up or not adhering to their follow-up visit schedule may have a higher proportion of detectable VLs. Due to varying lower limits of detection among the different VL analysers used, VL specimens <400 copies/mL were excluded from the analyses where median log_10_ values were required. Nevertheless, we used ratios of suppressed to detectable VLs to visualize changes in VL suppression rates over time.

Furthermore, demographic data was limited to laboratory-captured information and ART history was not captured. Although our results reveal a higher proportion of detectable HIV VL records from female patients, it is possible that this could be a bias of the demographics of the population accessing HIV care, and not necessarily an indication that more women are viraemic on ART. This in part could be associated with poor retention in HIV care among males as reported by the UNAIDS and as shown by studies from other African countries, including South Africa [55, 56]. Lastly, there is a need to prospectively assess longitudinal geospatial trends of VLs to assess whether the hotspots of VLs observed were the impact of the pandemic that settles over time as the health system recovers from the impact of COVID-19 pandemic, or whether this is a concerning trend that becomes sustained or worse over time.

## Conclusions

In conclusion, the application of geospatial analysis and visualization of the HIV epidemic by mapping routinely collected programmatic VL data, highlights potential weaknesses in healthcare facilities across KwaZulu-Natal province, South Africa. Identifying facilities with high proportions of people with low- and high-level viraemia and loci of at-risk populations provides invaluable data to both clinicians and policymakers, emphasizing the need for ongoing HIV VL monitoring coupled with HIV drug resistance surveillance. Such geospatial analyses allow for geographically targeted interventions in the HIV treatment programme, including directed improvement of health services and training of health professionals at specific facilities within identified hotspots in KZN, South Africa. Therefore, despite the rollout of DTG, there is a need for targeted deployment of public health resources in HIV hyperendemic regions, with the goal of enhancing HIV viral re-suppression in PLWH and at a public health level, preventing further HIV transmission and HIV-related deaths.

## Supporting information

S1 Fig Ratio of detectable to suppressed HIV viral loads across subdistricts in KwaZulu-Natal province, South Africa. The ratio of detectable (≥400 copies/mL) to suppressed (<400 copies/mL) HIV viral loads were mapped per facility using the inverse distance weighted interpolation method. Spectral colour change from blue to red reflects the increase in ratio of detectable to suppressed HIV viral loads. A ratio of 0,05 indicates 1 detectable viral load per 20 suppressed viral loads, while a ratio of 0,5 indicates 1 detectable viral load per 2 suppressed viral loads. Thick black outlines represent the borders of the 11 districts. Thin outlines represent the borders of the 44 subdistricts of KwaZulu-Natal. Republished from https://pinea.app.carto.com/map/4d4c56c1-f82d-4409-b190-ea9ced309005 under a CC BY license, with permission from Carto Builder user Lilishia Gounder, original copyright 2024.
(TIF)

S2 Fig Violin plot of HIV viral loads in KwaZulu-Natal province, South Africa, for the period 2018 to 2022. mL, millilitre; VL, viral load. The violin plot for log_10_ viral loads ≥400 copies/millilitre sampled across KwaZulu-Natal subdistricts showed a significant downward trend by year, p<0.01 Jonckheere–Terpstra test.
(TIF)

S3 Fig Linear prediction of median log10 HIV viral loads per facility in KwaZulu-Natal province, South Africa, for the period 2018 to 2022. VL, viral load. The linear prediction model for log_10_ viral loads ≥400 copies/millilitre sampled across KwaZulu-Natal subdistricts showed a significant downward trend by year, p<0.01 Jonckheere–Terpstra test.
(TIF)

S4 Fig Decreasing HIV viral loads across subdistricts in KwaZulu-Natal province, South Africa, between 2018 and 2022. Choropleth maps show median log_10_ viral loads ≥400 copies/millilitre sampled across KwaZulu-Natal subdistricts. Thick black outlines represent the borders of the 11 districts. Thin outlines represent the borders of the 44 subdistricts of KwaZulu-Natal. Republished from https://pinea.app.carto.com/map/4d4c56c1-f82d-4409-b190-ea9ced309005 under a CC BY license, with permission from Carto Builder user Lilishia Gounder, original copyright 2024.
(TIF)

S5 Fig HIV viral loads per facility before and during the transition to dolutegravir in KwaZulu-Natal province, South Africa. Inverse distance weighted interpolation maps showing changes in median log_10_ viral loads at the facility level. Map A represents the period before dolutegravir rollout i.e., 2018–2019 and map B represents the transition period to dolutegravir i.e., 2020–2022. Spectral colour change from red to blue reflects a decrease in median log_10_ viral loads. Republished from https://pinea.app.carto.com/map/4d4c56c1-f82d-4409-b190-ea9ced309005 under a CC BY license, with permission from Carto Builder user Lilishia Gounder, original copyright 2024.
(TIF)

S6 Fig Moran scatterplots of median log_10_ HIV viral loads per facility in KwaZulu-Natal province, South Africa, between 2018 and 2022. For cluster analysis, we used the median log_10_ HIV viral load per facility to calculate Moran’s I statistic and p-values, and created Moran scatterplots for each year of the study period. Each plot represents an individual year: (A) 2018, (B) 2019, (C) 2020, (D) 2021 and (E) 2022. Moran’s I statistic measures spatial autocorrelation and index values for Moran’s I statistic range from -1 to +1. A positive index value indicates positive spatial autocorrelation.
(TIF)

S1 Table Proportion of all HIV viral loads by year and district. mL, millilitre; VL, viral load. The proportion of VL records collected per facility is cumulatively represented as a proportion per district and grouped according to the district in which that facility is located.
(DOCX)

S2 Table Proportion of HIV viral loads by district and year, from hotspot facilities demonstrating spatial clustering of high median log_10_ HIV viral loads. VL, viral load. ^a^ 244 unique hotspot facilities demonstrating spatial clustering of high median log_10_ HIV VLs were identified from 2018 to 2022 cumulatively. However, some facilities were repeatedly identified as hotspot facilities and were therefore included in the different analyses for each of those specific years. ^b^ Each unique viral load record was included in the analysis based on the status of the facility and the specific year in which it was collected. If facilities were identified as statistically significant hotspots based on p<0.05 Getis-Ord Gi* statistic, the viral load records linked to that facility and year were included in the respective analysis. ^c^ Hotspot facilities were identified in 10 of the 11 districts. Facilities from uThukela did not have spatial clustering of high median log_10_ HIV VLs during the study period. The proportion of VL records per hotspot facility is cumulatively represented as a proportion per district, grouped according to the district in which that facility is located.
(DOCX)

S3 Table Comparison of facilities within hot- and coldspots from 2018 to 2022. IQR, interquartile range; mL, millilitre; VL, viral load. ^a^ Each unique viral load record was included in the analysis based on the status of the facility and the specific year in which it was collected. If facilities were identified as statistically significant hotspots or coldspots based on p<0.05 Getis-Ord Gi* statistic, the viral load records linked to that facility and year were included in the respective analysis. ^b^ Records from correctional facilities or frail care facilities. ^c^ Obtained from annual reports and integrated development plans for individual subdistricts.
(DOCX)

S1 Data Minimal dataset. (XLSX)
