# Supplementary figures and images for: Geospatial and temporal mapping of detectable HIV-1 viral loads amid dolutegravir rollout in KwaZulu-Natal, South Africa
Source: PLOS Glob Public Health. 2024 May 28;4(5):e0003224. doi: 10.1371/journal.pgph.0003224 (PMC11132473; doi:10.1371/journal.pgph.0003224)

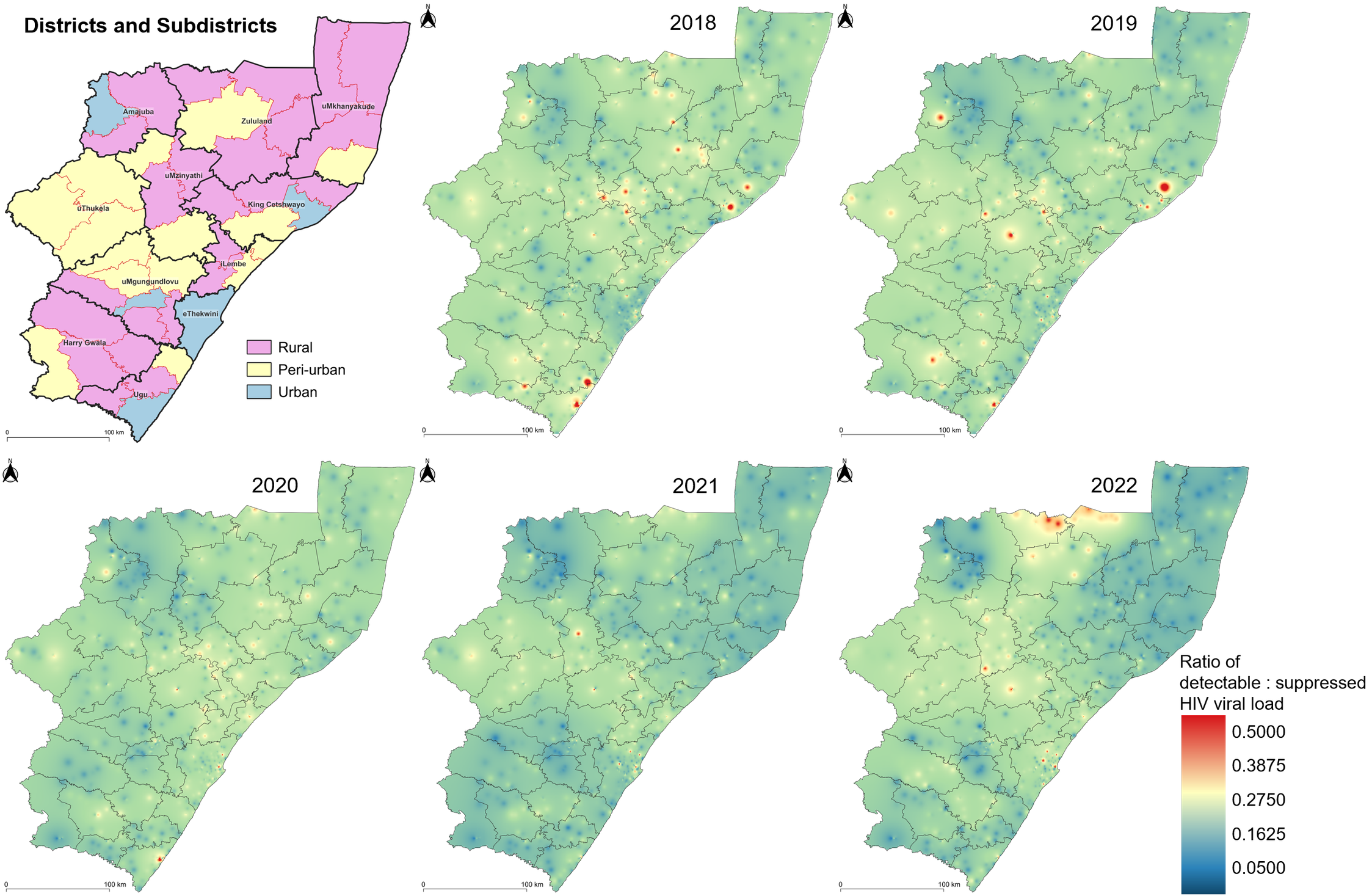

Supplement: S1 Fig — The ratio of detectable (≥400 copies/mL) to suppressed (<400 copies/mL) HIV viral loads were mapped per facility using the inverse distance weighted interpolation method. Spectral colour change from blue to red reflects the increase in ratio of detectable to suppressed HIV viral loads. A ratio of 0,05 indicates 1 detectable viral load per 20 suppressed viral loads, while a ratio of 0,5 indicates 1 detectable viral load per 2 suppressed viral loads. Thick black outlines represent the borders of the 11 districts. Thin outlines represent the borders of the 44 subdistricts of KwaZulu-Natal. Republished from https://pinea.app.carto.com/map/4d4c56c1-f82d-4409-b190-ea9ced309005 under a CC BY license, with permission from Carto Builder user Lilishia Gounder, original copyright 2024. (TIF) [file pgph.0003224.s001.tif]

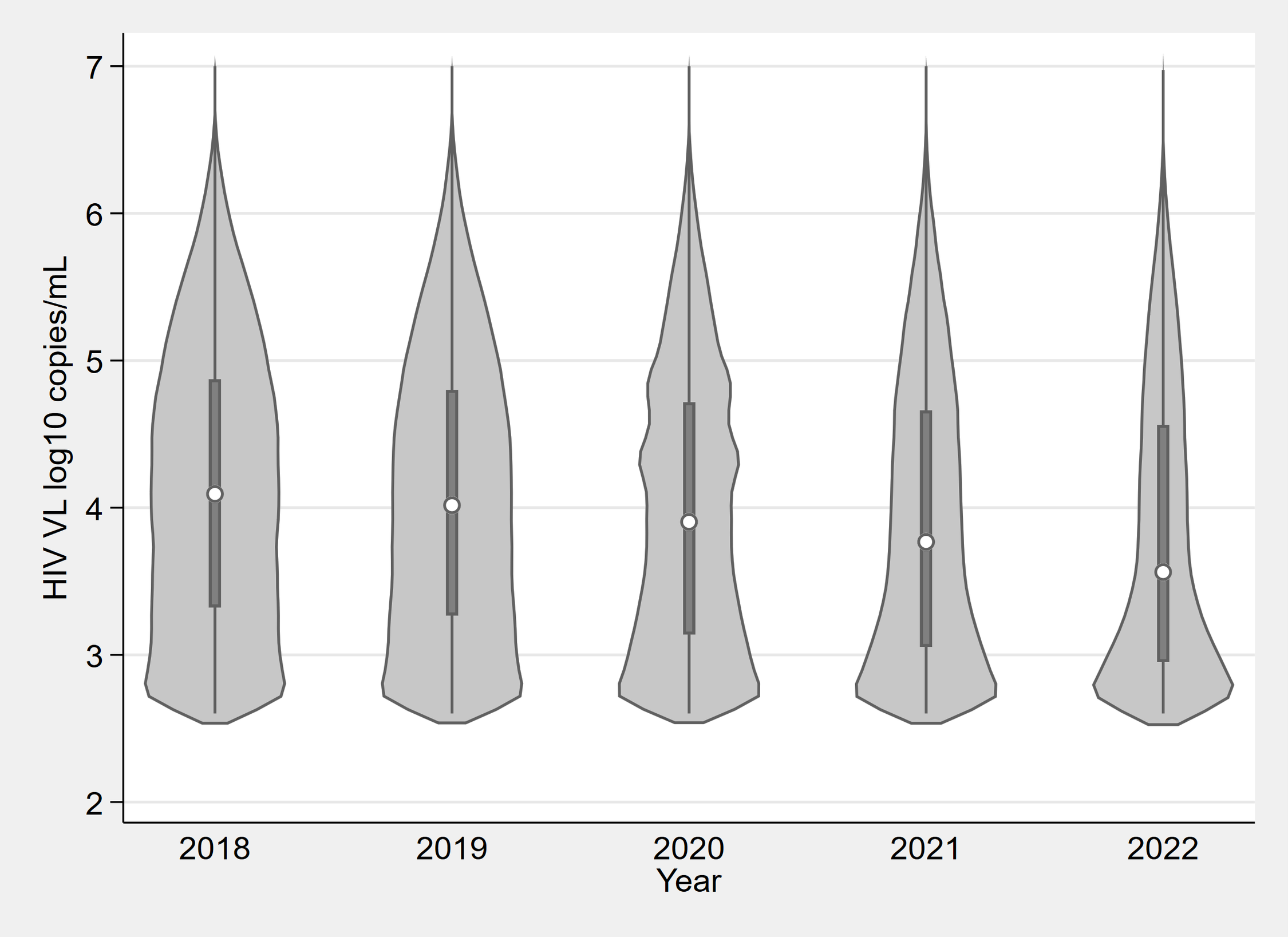

Supplement: S2 Fig — mL, millilitre; VL, viral load. The violin plot for log10 viral loads ≥400 copies/millilitre sampled across KwaZulu-Natal subdistricts showed a significant downward trend by year, p<0.01 Jonckheere–Terpstra test. (TIF) [file pgph.0003224.s002.tif]

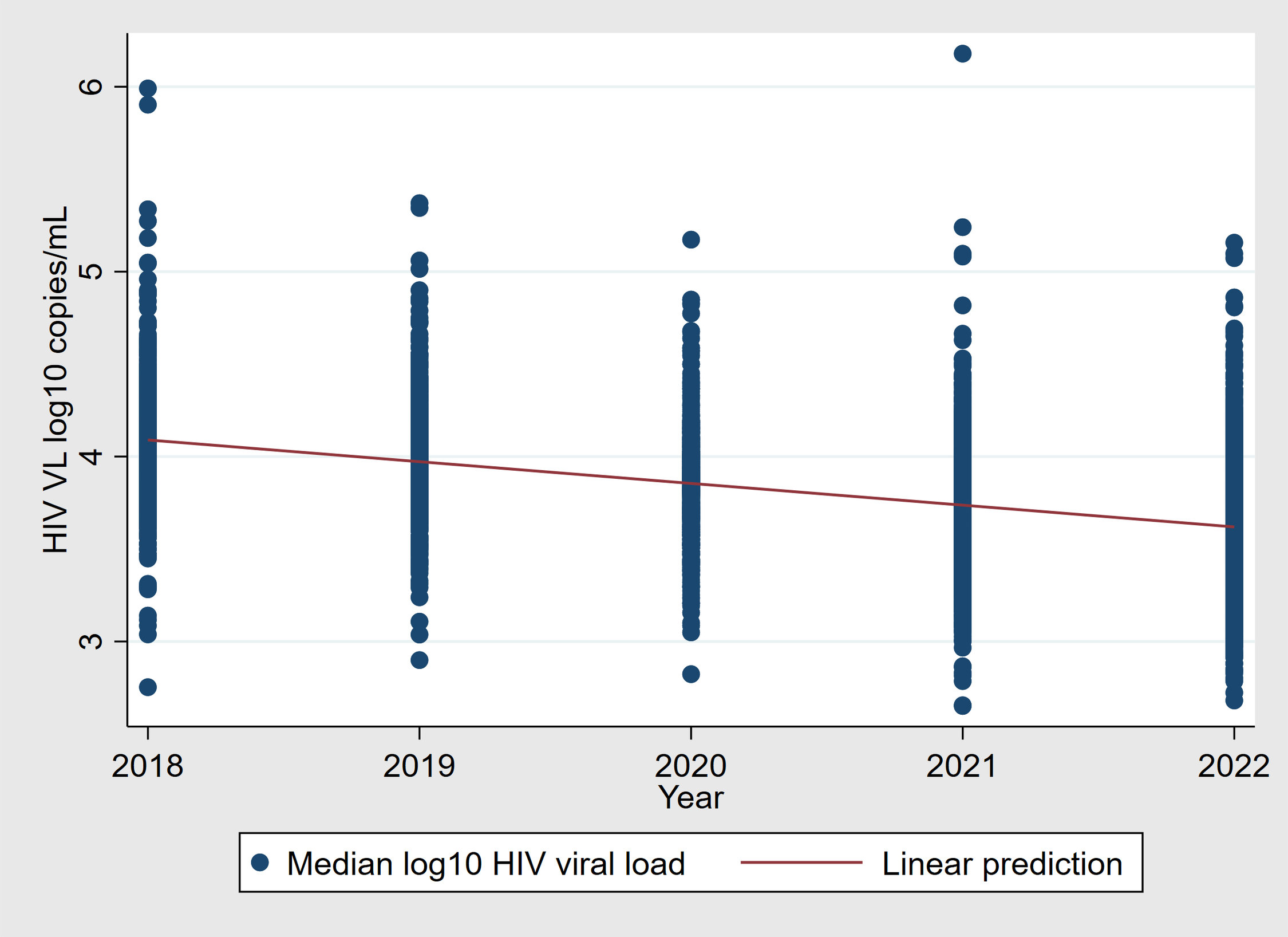

Supplement: S3 Fig — VL, viral load. The linear prediction model for log10 viral loads ≥400 copies/millilitre sampled across KwaZulu-Natal subdistricts showed a significant downward trend by year, p<0.01 Jonckheere–Terpstra test. (TIF) [file pgph.0003224.s003.tif]

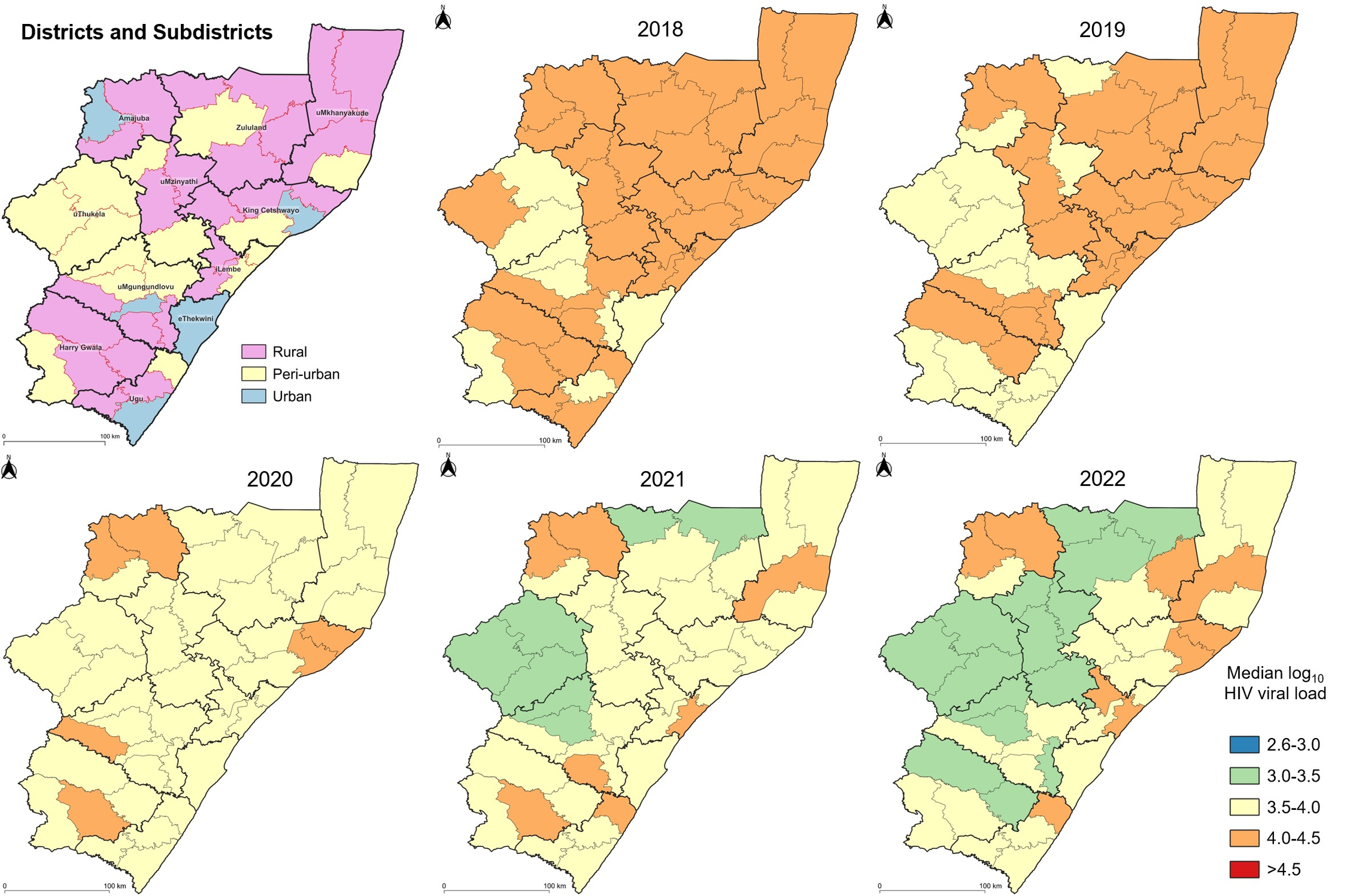

Supplement: S4 Fig — Choropleth maps show median log10 viral loads ≥400 copies/millilitre sampled across KwaZulu-Natal subdistricts. Thick black outlines represent the borders of the 11 districts. Thin outlines represent the borders of the 44 subdistricts of KwaZulu-Natal. Republished from https://pinea.app.carto.com/map/4d4c56c1-f82d-4409-b190-ea9ced309005 under a CC BY license, with permission from Carto Builder user Lilishia Gounder, original copyright 2024. (TIF) [file pgph.0003224.s004.tif]

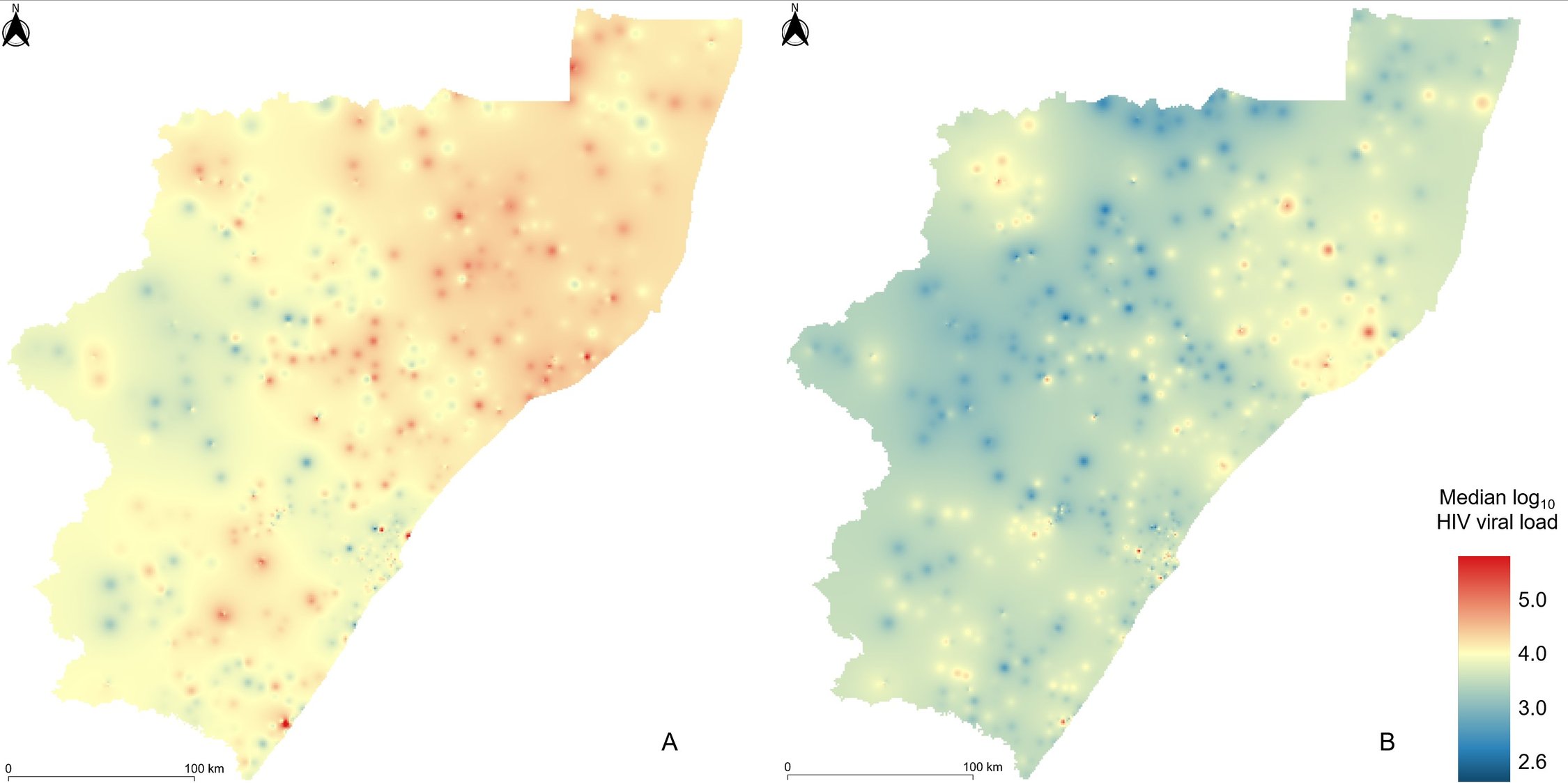

Supplement: S5 Fig — Inverse distance weighted interpolation maps showing changes in median log10 viral loads at the facility level. Map A represents the period before dolutegravir rollout i.e., 2018–2019 and map B represents the transition period to dolutegravir i.e., 2020–2022. Spectral colour change from red to blue reflects a decrease in median log10 viral loads. Republished from https://pinea.app.carto.com/map/4d4c56c1-f82d-4409-b190-ea9ced309005 under a CC BY license, with permission from Carto Builder user Lilishia Gounder, original copyright 2024. (TIF) [file pgph.0003224.s005.tif]

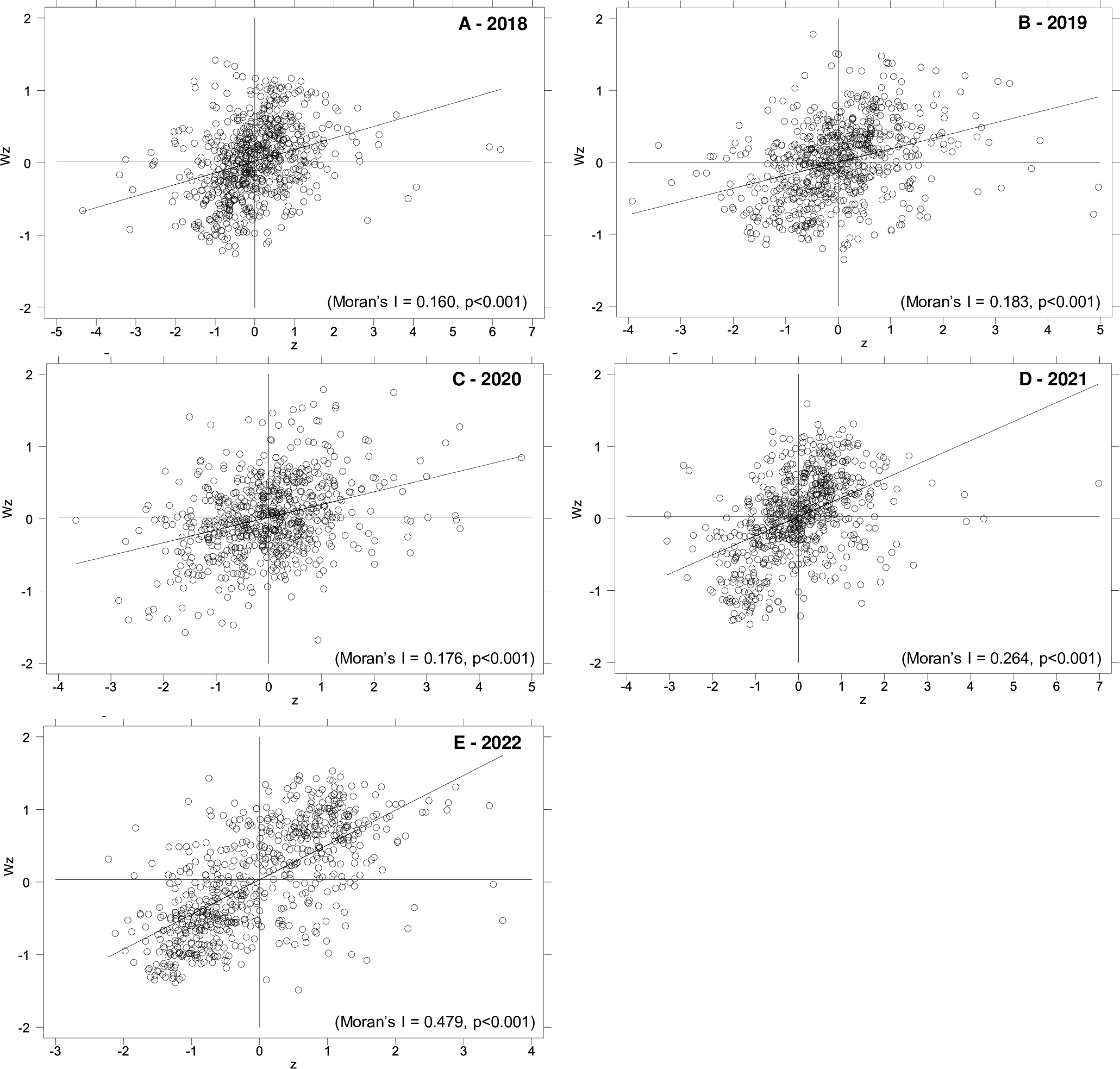

Supplement: S6 Fig — For cluster analysis, we used the median log10 HIV viral load per facility to calculate Moran’s I statistic and p-values, and created Moran scatterplots for each year of the study period. Each plot represents an individual year: (A) 2018, (B) 2019, (C) 2020, (D) 2021 and (E) 2022. Moran’s I statistic measures spatial autocorrelation and index values for Moran’s I statistic range from -1 to +1. A positive index value indicates positive spatial autocorrelation. (TIF) [file pgph.0003224.s006.tif]
