# Supplementary material for: Geospatial and temporal mapping of detectable HIV-1 viral loads amid dolutegravir rollout in KwaZulu-Natal, South Africa
Source: PLOS Glob Public Health. 2024 May 28;4(5):e0003224. doi: 10.1371/journal.pgph.0003224 (PMC11132473; doi:10.1371/journal.pgph.0003224)
Supplement: S1 Table — mL, millilitre; VL, viral load. The proportion of VL records collected per facility is cumulatively represented as a proportion per district and grouped according to the district in which that facility is located. (DOCX) [file pgph.0003224.s007.docx]

| **District** |  | **2018** | | **2019** | | **2020** | | **2021** | | **2022** | |
| --- | --- | --- | --- | --- | --- | --- | --- | --- | --- | --- | --- |
|  | VL in copies/mL | ≥400 | <400 | ≥400 | <400 | ≥400 | <400 | ≥400 | <400 | ≥400 | <400 |
| **Amajuba** | n (%) | 7 061 (12.32) | 50 258 (87.68) | 7 575 (10.95) | 61 618 (89.05) | 8 111 (11.10) | 64 930 (88.90) | 7 175 (9.72) | 66 632 (90.28) | 3 558 (9.28) | 34 777 (90.72) |
| **eThekwini** | n (%) | 59 501 (11.89) | 440 837 (88.11) | 64 588 (12.24) | 463 188 (87.76) | 80 485 (14.36) | 479 908 (85.64) | 69 768 (12.26) | 499 531 (87.74) | 43 922 (14.17) | 266 131 (85.83) |
| **Harry Gwala** | n (%) | 9 845 (15.99) | 51 724 (84.01) | 11 161 (15.97) | 58 743 (84.03) | 10 137 (13.19) | 66 719 (86.81) | 9 655 (12.77) | 65 977 (87.23) | 5 706 (14.15) | 34 610 (85.85) |
| **iLembe** | n (%) | 14 249 (16.33) | 73 000 (83.67) | 14 801 (15.48) | 80 810 (84.52) | 16 710 (17.37) | 79 505 (82.63) | 15 639 (15.29) | 86 663 (84.71) | 7 589 (14.27) | 45 605 (85.73) |
| **King Cetshwayo** | n (%) | 21 198 (13.90) | 131 285 (86.10) | 21 983 (13.76) | 137 835 (86.24) | 24 758 (14.27) | 148 728 (85.73) | 19 448 (11.45) | 150 341 (88.55) | 10 445 (11.45) | 80 809 (88.55) |
| **Ugu** | n (%) | 18 054 (15.09) | 101 626 (84.91) | 16 617 (13.43) | 107 158 (86.57) | 17 036 (12.90) | 115 077 (87.10) | 15 513 (11.89) | 114 999 (88.11) | 7 996 (11.81) | 59 699 (88.19) |
| **uMgungundlovu** | n (%) | 21 222 (13.47) | 136 376 (86.53) | 25 838 (14.69) | 150 055 (85.31) | 24 394 (12.93) | 164 223 (87.07) | 22 662 (11.84) | 168 766 (88.16) | 13 332 (12.88) | 90 140 (87.12) |
| **uMkhanyakude** | n (%) | 15 115 (13.30) | 98 534 (86.70) | 15 055 (12.65) | 103 930 (87.35) | 17 541 (13.93) | 108 381 (86.07) | 14 149 (11.46) | 109 353 (88.54) | 6 831 (10.11) | 60 765 (89.89) |
| **uMzinyathi** | n (%) | 9 711 (15.30) | 53 756 (84.70) | 10 046 (14.70) | 58 311 (85.30) | 9 827 (13.80) | 61 387 (86.20) | 10 448 (14.52) | 61 515 (85.48) | 6 721 (17.07) | 32 646 (82.93) |
| **uThukela** | n (%) | 15 708 (16.51) | 79 448 (83.49) | 18 935 (17.48) | 89 386 (82.52) | 15 831 (13.82) | 98 682 (86.18) | 18 869 (15.43) | 103 423 (84.57) | 12 120 (16.70) | 60 438 (83.30) |
| **Zululand** | n (%) | 16 860 (15.35) | 92 948 (84.65) | 17 068 (13.94) | 105 330 (86.06) | 20 633 (15.08) | 116 164 (84.92) | 19 122 (13.75) | 119 952 (86.25) | 12 849 (16.26) | 66 175 (83.74) |

**S1 Table. Proportion of all HIV viral loads by year and district.**

mL, millilitre; VL, viral load

The proportion of VL records collected per facility is cumulatively represented as a proportion per district and grouped according to the district in which that facility is located.
