# Supplementary material for: Geospatial and temporal mapping of detectable HIV-1 viral loads amid dolutegravir rollout in KwaZulu-Natal, South Africa
Source: PLOS Glob Public Health. 2024 May 28;4(5):e0003224. doi: 10.1371/journal.pgph.0003224 (PMC11132473; doi:10.1371/journal.pgph.0003224)
Supplement: S2 Table — VL, viral load. a 244 unique hotspot facilities demonstrating spatial clustering of high median log10 HIV VLs were identified from 2018 to 2022 cumulatively. However, some facilities were repeatedly identified as hotspot facilities and were therefore included in the different analyses for each of those specific years. b Each unique viral load record was included in the analysis based on the status of the facility and the specific year in which it was collected. If facilities were identified as statistically significant hotspots based on p<0.05 Getis-Ord Gi* statistic, the viral load records linked to that facility and year were included in the respective analysis. c Hotspot facilities were identified in 10 of the 11 districts. Facilities from uThukela did not have spatial clustering of high median log10 HIV VLs during the study period. The proportion of VL records per hotspot facility is cumulatively represented as a proportion per district, grouped according to the district in which that facility is located. (DOCX) [file pgph.0003224.s008.docx]

|  | **2018** | **2019** | **2020** | **2021** | **2022** |
| --- | --- | --- | --- | --- | --- |
| Number of facilities ^a^, (number of VL records) ^b^ | 69  (18 045) | 69  (19 460) | 55  (21 826) | 81  (27 263) | 175  (28 912) |
| **District** n, (%) ^c^ |  |  |  |  |  |
| Amajuba | 0 (0.00) | 0 (0.00) | 2 321 (10.63) | 6 784 (24.88) | 2 376 (8.22) |
| eThekwini | 0 (0.00) | 0 (0.00) | 7 096 (32.51) | 660 (2.42) | 4 746 (16.42) |
| Harry Gwala | 0 (0.00) | 246 (1.26) | 284 (1.30) | 230 (0.84) | 0 (0.00) |
| iLembe | 0 (0.00) | 0 (0.00) | 0 (0.00) | 6 370 (23.37) | 4 986 (17.25) |
| King Cetshwayo | 4 323 (23.96) | 13.796 (70.89) | 12 125 (55.55) | 7 163 (26.27) | 6 265 (21.67) |
| Ugu | 5 854 (32.44) | 0 (0.00) | 0 (0.00) | 0 (0.00) | 985 (3.41) |
| uMgungundlovu | 427 (2.37) | 1 088 (5.59) | 0 (0.00) | 1 839 (6.75) | 2 813 (9.73) |
| uMkhanyakude | 294 (1.63) | 0 (0.00) | 0 (0.00) | 954 (3.50) | 3 946 (13.65) |
| uMzinyathi | 230 (1.27) | 190 (0.98) | 0 (0.00) | 0 (0.00) | 0 (0.00) |
| Zululand | 6 917 (38.33) | 4 140 (21.27) | 0 (0.00) | 3 263 (11.97) | 2 795 (9.67) |

**S2 Table. Proportion of HIV viral loads by district and year, from hotspot facilities demonstrating spatial clustering of high median log_10_ HIV viral loads.** VL, viral load

^a^ 244 unique hotspot facilities demonstrating spatial clustering of high median log_10_ HIV VLs were identified from 2018 to 2022 cumulatively. However, some facilities were repeatedly identified as hotspot facilities and were therefore included in the different analyses for each of those specific years.

^b^ Each unique viral load record was included in the analysis based on the status of the facility and the specific year in which it was collected. If facilities were identified as statistically significant hotspots based on p<0.05 Getis-Ord Gi* statistic, the viral load records linked to that facility and year were included in the respective analysis.

^c^ Hotspot facilities were identified in 10 of the 11 districts. Facilities from uThukela did not have spatial clustering of high median log_10_ HIV VLs during the study period. The proportion of VL records per hotspot facility is cumulatively represented as a proportion per district, grouped according to the district in which that facility is located.
