# Supplementary material for: Geospatial and temporal mapping of detectable HIV-1 viral loads amid dolutegravir rollout in KwaZulu-Natal, South Africa
Source: PLOS Glob Public Health. 2024 May 28;4(5):e0003224. doi: 10.1371/journal.pgph.0003224 (PMC11132473; doi:10.1371/journal.pgph.0003224)
Supplement: S3 Table — IQR, interquartile range; mL, millilitre; VL, viral load. a Each unique viral load record was included in the analysis based on the status of the facility and the specific year in which it was collected. If facilities were identified as statistically significant hotspots or coldspots based on p<0.05 Getis-Ord Gi* statistic, the viral load records linked to that facility and year were included in the respective analysis. b Records from correctional facilities or frail care facilities. c Obtained from annual reports and integrated development plans for individual subdistricts. (DOCX) [file pgph.0003224.s009.docx]

**S3 Table.** Comparison of facilities within hot- and coldspots from 2018 to 2022

|  | **2018** | | **2019** | | **2020** | | **2021** | | **2022** | |
| --- | --- | --- | --- | --- | --- | --- | --- | --- | --- | --- |
| **Variable** | **Hotspot** | **Coldspot** | **Hotspot** | **Coldspot** | **Hotspot** | **Coldspot** | **Hotspot** | **Coldspot** | **Hotspot** | **Coldspot** |
| Number of facilities, | 69 | 63 | 69 | 78 | 55 | 49 | 81 | 79 | 175 | 127 |
| (number of VL records)^a^ | (18 045) | (28 710) | (19 460) | (30 789) | (21 826) | (11 951) | (27 263) | (29 776) | (28 912) | (32 224) |
| **Sex** |  |  |  |  |  |  |  |  |  |  |
| Male | 6 333 (35.10%) | 10 543 (36.72%) | 7 136 (36.67%) | 10 748 (34.91%) | 7 501 (34.37%) | 4 340 (36.31%) | 9 499 (34.84%) | 10 459 (35.13%) | 10 161 (35.14%) | 11 397 (35.37%) |
| Female | 11 249 (62.34%) | 17 505 (60.97%) | 11 653 (59.88%) | 19 338 (62.81%) | 13 618 (62.39%) | 7 390 (61.84%) | 16 583 (60.83%) | 18 301 (61.46%) | 17 709 (61.25%) | 19 902 (61.76%) |
| Unknown | 463 (2.57%) | 662 (2.31%) | 671 (3.45%) | 703 (2.28%) | 707 (3.24%) | 221 (1.85%) | 1 181 (4.33 %) | 1 016 (3.41%) | 1 042 (3.60%) | 925 (2.87%) |
| **Age** in years,  median (IQR) | 32  (23-39) | 33  (24-41) | 33  (24-41) | 34  (25-42) | 33  (25-40) | 33  (22-43) | 33  (24-41) | 35  (25-44) | 34  (24-42) | 37  (27-45) |
| <5 | 313 (1.73%) | 481 (1.68%) | 250 (1.28%) | 393 (1.28%) | 416  (1.91%) | 122  (1.02%) | 509 (1.87%) | 362 (1.22%) | 483 (1.67%) | 308 (0.96%) |
| 5-14 | 1 556 (8.62%) | 2 201 (7.67%) | 1 319 (6.78%) | 2 048 (6.65%) | 1 202  (5.51%) | 1 151  (9.63%) | 1 734 (6.36%) | 2 299 (7.72%) | 1 813 (6.27%) | 1 894 (5.88%) |
| 15-24 | 3 168 (17.56%) | 4 541 (15.82%) | 3 320 (17.06%) | 4 648 (15.10%) | 3 298  (15.11%) | 2 210  (18.49%) | 4 786 (17.55%) | 4 591 (15.42%) | 4 914 (17.00%) | 4 095 (12.71%) |
| 25-49 | 11 404 (63.20%) | 18 730 (65.24%) | 12 571 (64.60%) | 20 348 (66.09%) | 14 797  (67.80%) | 6 897  (57.71%) | 17 109 (62.76%) | 18 169 (61.02%) | 18 185 (62.90%) | 20 482 (63.56%) |
| >50 | 1 402 (7.77%) | 2 588 (9.01%) | 1 783 (9.16%) | 3 090 (10.04%) | 1 823  (8.35%) | 1 499  (12.54%) | 2 692 (9.87%) | 4 043 (13.58%) | 3 208 (11.10%) | 5 160 (16.01%) |
| Unknown | 202 (1.12%) | 169 (0.59%) | 217 (1.12%) | 262 (0.85%) | 290  (1.33%) | 72  (0.60%) | 433 (1.59%) | 312 (1.05%) | 309 (1.07%) | 285 (0.88%) |
| **VL log_10_** copies/mL,  median (IQR) | 4.267 (3.455-5.041) | 3.883 (3.188-4.673) | 4.250 (3.436-5.009) | 3.818 (3.143-4.612) | 4.090 (3.276-4.849) | 3.627 (3.017-4.566) | 4.072 (3.258-4.894) | 3.368 (2.904-4.324) | 4.08 (3.267-4.919) | 3.196 (2.848-4.086) |
| **Healthcare department** |  |  |  |  |  |  |  |  |  |  |
| Outpatients | 16 556 (91.75%) | 26 526 (92.39%) | 17 750 (91.21%) | 29 135 (94.63%) | 19 249 (88.19%) | 11 076 (92.68%) | 23 309 (85.50%) | 27 445 (92.17%) | 25 221 (87.23%) | 30 609 (94.99%) |
| Inpatients | 1 451 (8.04%) | 2 163 (7.53%) | 1 654 (8.50%) | 1 612 (5.24%) | 2 470 (11.32%) | 855 (7.15%) | 3 869 (14.19%) | 2 260 (7.59%) | 3 661 (12.66%) | 1 540 (4.78%) |
| Other^b^ | 38 (0.21%) | 21 (0.07%) | 56 (0.29%) | 42 (0.14%) | 107 (0.49%) | 20 (0.17%) | 85 (0.31%) | 71 (0.24%) | 30 (0.10%) | 75 (0.23%) |
| **Level of urbanization**^c^ |  |  |  |  |  |  |  |  |  |  |
| Rural subdistricts | 7 702 (42.68%) | 394  (1.37%) | 8 351 (42.91%) | 2 172 (7.05%) | 1 974 (9.04%) | 5 953 (49.81%) | 5 302 (19.45%) | 6 956 (23.36%) | 8 341 (28.85%) | 6 973 (21.64%) |
| Peri-urban subdistricts | 1 298 (7.19%) | 12 520 (43.61%) | 809 (4.16%) | 16 386 (53.22%) | 567 (2.60%) | 5 998 (50.19%) | 7 771 (28.50%) | 18 660 (62.67%) | 6 430 (22.24%) | 16 603 (51.52%) |
| Urban subdistricts | 9 045 (50.12%) | 15 796 (55.02%) | 10 300 (52.93%) | 12 231 (39.73%) | 19 285 (88.36%) | 0  (0.00%) | 14 190 (52.05%) | 4 160 (13.97%) | 14 141 (48.91%) | 8 648 (26.84%) |

IQR, interquartile range; mL, millilitre; VL, viral load

^a^ Each unique viral load record was included in the analysis based on the status of the facility and the specific year in which it was collected. If facilities were identified as statistically significant hotspots or coldspots based on p<0.05 Getis-Ord Gi* statistic, the viral load records linked to that facility and year were included in the respective analysis.

^b^ Records from correctional facilities or frail care facilities.

^c^ Obtained from annual reports and integrated development plans for individual subdistricts**.**
